# Surgical Site Infections and Perioperative Optimization of Host Immunity by Selection of Anesthetics

**DOI:** 10.1155/2021/5576959

**Published:** 2021-03-08

**Authors:** Koichi Yuki, Miho Shibamura-Fujiogi

**Affiliations:** ^1^Department of Anesthesiology, Perioperative and Pain Medicine, Cardiac Anesthesia Division, Boston Children's Hospital, 300 Longwood Avenue, Boston, MA 02115, USA; ^2^Department of Anaesthesia, Harvard Medical School, USA; ^3^Department of Immunology, Harvard Medical School, USA

## Abstract

Surgical site infections are significant health care issues, and efforts to mitigate their occurrence have been ongoing worldwide, mainly focusing to reduce the spillage of microbes to the otherwise sterile tissues. Optimization of host immunity has been also recognized including temperature regulation (normothermia), adequate oxygenation, and glucose management. A number of papers have described the role of anesthetics in host immunity. The role of anesthetics in postoperative outcomes including surgical site infections has been also studied. We will review the current literature and propose the importance of anesthetic selection to potentially mitigate surgical site infections.

## 1. Introduction

Forty-five million inpatient surgical procedures are performed annually in the United States. Surgical site infections (SSIs), previously called surgical wound infections, are the most common nosocomial infections in surgical patients, occupying approximately 20% of the estimated two million nosocomial infections in the United States and responsible for the aggregate annual cost of $3.5-$10.1 billion [1]. When compared the estimated cost of the five most significant nosocomial infections, SSIs were most responsible [2]. These statistics may underestimate the incidence and cost of SSIs, because more than 34 million surgical procedures in outpatient ambulatory surgical centers are not included in these figures [3]. SSIs are associated with significant morbidities and mortalities; SSIs are associated with increased readmission and length of hospital stay [4]. Death was seen twice more in patients with SSI than those who do not have SSI [5]. Thus, every effort should be made to reduce the incidence of SSIs. World Health Organization (WHO) has published the recommendations of perioperative management of surgical patients to reduce SSIs in 2016 [6, 7]. However, they remain to be a significant public health issue.

Surgical procedures involve the incision and dissection of tissues, which can introduce microbes to the otherwise sterile sites. Adequate immunity is critical as a self-defense machinery for patients to fight against invading microbes, while antibiotic administration also helps to reduce microbial burden. The reports that perioperative anesthetics can be immunomodulatory so as to affect postoperative outcomes have been increasingly recognized [8–11]. Here, we will review the current knowledge about the effect of anesthetics on host immune functions and SSIs.

## 2. Methods

### 2.1. Database Search

We searched electronic databases for anesthetic effect on immune cells: MEDLINE/PubMed until October 30, 2020. We focused to identify studies describing the effect of volatile anesthetics on neutrophils and monocytes/macrophages, surgical site infections/sepsis. “Isoflurane,” “Sevoflurane,” “Desflurane,” “Halothane,” “Ether,” “Propofol,” “Volatile anesthetics,” “Inhaled anesthetics,” “Intravenous anesthetics,” “Anesthesia,” “Neutrophil,” “Monocyte,” “Macrophage,” “Surgical site infections,” “Wound infections,” and “Sepsis” were chosen for search.

## 3. Clinical Data of Surgical Site Infections: Diagnosis, Statistics, and SSI Bundles

SSI is defined as an infection occurring within 30 days after a surgical operation (or within 90 days after an implant is left in place after the procedure) by the Centers for Disease Control and Prevention (CDC)'s National Healthcare Surveillance Network (NHSN) [12]. SSI is classified as either incisional or organ/space-related. Incisional SSI is further classified as superficial (skin or subcutaneous tissue) or deep (fascia and/or muscle layer). Organ/space SSIs include infections in tissues which are deep to the fascia and opened or manipulated during surgery. Superficial SSIs require less aggressive interventions. In contrast, deep/organ-space SSIs often require invasive management including the administration of intravenous antibiotics, percutaneous drainage procedures, and reoperations [13]. The majority of rates of SSIs reportedly range from 2-3% to as high as 20% [14, 15].

Wounds can be classified into (1) clean, (2) clean-contaminated, (3) contaminated, and (4) dirty wounds. In most SSIs, responsible pathogens originate from patient's endogenous flora [16]. Based on the NHSN data, *Staphylococcus aureus* (*S. aureus*), *Enterococcus faecalis* (*E. faecalis*), and *Escherichia coli* (*E. coli*) are the most common microbial species involved in SSIs [17], but pathogens isolated from infection sites highly depend on the type of procedures [15]. Wounds are often polymicrobial [18]. Methicillin-resistant *Staphylococcus aureus* (MRSA) infection is most frequently encountered among pathogens responsible for SSIs. However, microbes seen in surgical wounds is also dependent on the type of surgical procedure. *E. coli* and *Bacteroides fragilis* (*B. fragilis*) are the most common organisms in colon surgery [19]. Without surprise, the nature of wounds is strongly associated with the incidence of SSIs. In one study, clean, clean-contaminated, contaminated, and dirty wounds were associated with the SSI rate of 2.6%, 6.7%, 8.6%, and 11.8%, respectively [20]. SSIs are particularly common for colorectal procedures with the incidence ranging from 5% to 26% [13, 21–24].

Because of the awareness of seriousness of SSIs, reducing SSI rates became a national priority project in early 2000. The Surgical Care Improvement Project (SCIP) was implemented by the Centers for Medicare and Medicaid Services in 2006. SCIP infection prevention measures for colorectal surgery included (1) appropriate, prophylactic antibiotic selection and its administration within 60 minutes before surgical incision, (2) discontinuation of prophylactic antibiotic within 24 hours after surgery, (3) appropriate hair removal, and (4) maintenance of normothermia (postoperative temperature > 36°C). However, the adherence to the clinical bundle did not improve the incidence of SSIs [25]. To better control SSIs, a number of local quality improvement (QI) programs were initiated. The Comprehensive Unit-Based Safety Program (CUSP) was a statewide QI collaborative in Michigan [26]. The items in the bundle included (1) administration of preoperative chlorhexidine shower, (2) preoperative mechanical bowel preparation with oral antibiotics, (3) warming of patients in the preanesthesia area, (4) standardization of skin preparation, (5) standardized adaptation of prophylactic antibiotics, and (6) enhancement of intraoperative sterile techniques. The bundle proposed in Rhode Island was similar, containing (1) preoperative bowel preparation with oral antibiotics, (2) hair removal using clippers, (3) preoperative glucose check and control, (4) prophylactic antibiotic administration within 60 minutes before surgical incision, and redosing of cases >3 hours, (5) intraoperative inspired oxygen concentration (FiO_2_) >0.6, (6) maintenance of intraoperative temperature > 36.0°C, (7) enhancement of intraoperative sterile techniques, and (8) administration of 100% nonrebreather face mask in the recovery room [27]. The initiation of both bundles reduced the incidence of SSIs. Incorporating the results from numerous studies, WHO issued the recommendations on preoperative, intraoperative, and postoperative measures to prevent SSIs [6, 7]. Recommendations were categorized either strong (the expert panel was confident that the benefits of the intervention outweighed the risks) or conditional (the panel considered that the benefits of the intervention probably outweighed the risks). Because “conditional” recommendations were based on very low evidence, we summarized the recommendations listed as “strong” in Table 1. The majority of recommendations primarily focus on reducing microbial contamination and burden. Despite general bundle approaches and recommendations, SSIs still remains to be a problem, and further effort is needed to mitigate SSIs. Here, we will review the role of host immune functions and SSIs, particularly in the context of perioperative anesthetics.

## 4. Host Immunological Responses to Surgery

Several risk factors of SSIs have been suggested, though they have not been validated in randomized controlled studies; preexisting infection, old age, smoking, ischemia secondary to vascular diseases, diabetes mellitus (DM), obesity, and the type and duration of surgical procedures [28], most of which involve patients' factors related to immune functions, indicating their importance in SSIs.

The immunological alternation in the perioperative setting derives from a combination of local and central events. A surgical insult initiates a series of immunological responses, which are largely divided into two phases: (1) proinflammatory responses to eradicate any causative microbes and secondary opportunistic ones and (2) systemic deactivation of the immune system to restore homeostasis. Adequate immunological responses protect against infection and provide effective wound healing, both of which are key determinants of smooth postoperative recovery. Phagocytes such as neutrophils, monocytes, macrophages, and dendritic cells protect the host as the front-line defense cells to ingest harmful particles and microbes.

The underlying mechanism of initial proinflammatory responses is as follows: Surgical dissection and ischemia-reperfusion can cause the necrosis of cells, which release danger signals as host molecules called damage-associated molecular pattern molecules (DAMPs) [29, 30]. DAMPs include high-mobility group box 1 (HMGB1) [31] and mitochondria DNA (mtDNA) [32] stimulate innate immune cells such as neutrophils and macrophages/monocytes to produce proinflammatory cytokines. DAMPs are recognized by pattern-recognition receptors (PRRs) on phagocytes to induce the production of proinflammatory cytokines such as tumor necrosis factor (TNF)-*α*, interleukin (IL)-1*β*, IL-6, and IL-8 in the presence of DAMPs [33–35]. Toll-like receptors (TLRs) are major PRRs that recognize a number of DAMPs including HMGB-1 and mtDNA as well as invading (and preexisting) microbes. Together with DAMPs/microbes, cytokines activate and recruit neutrophils and monocytes to inflammatory sites by interacting with cytokine receptors and TLRs [36, 37]. In addition to this local response, surgical insult stimulates the hypothalamic-pituitary-adrenal (HPA) axis and the sympathomedullary (SAM) axis via the afferent nerves to lead to the systemic secretion of cortisol and catecholamines. Anti-inflammatory responses occur in response to stress hormones cortisol and catecholamines; glucocorticoid receptors are expressed in neutrophils, monocytes, macrophages, T cells, and B cells. Cortisol shifts them to the cells with anti-inflammatory phenotype [38]. Catecholamine receptors are also found in neutrophils, monocytes, macrophages, natural killer (NK) cells, B cells, and T cells, and their stimulation induces anti-inflammatory responses [39]. Catecholamine-mediated anti-inflammatory responses are induced most potently by epinephrine, followed by norepinephrine, and least by cortisol [40]. Anti-inflammatory cytokines such as IL-10 and transforming growth factor (TGF)-*β* induce regulatory T cells, a subset of cluster of differentiation (CD) 4^+^ T cells with suppressive activity, from a pool of CD4^+^ T cells [41]. These regulatory T cells also bias CD4^+^ T cells toward anti-inflammatory Th2 cells [42]. In addition, DAMP members heat shock proteins (HSPs) and chaperone proteins released under stress, amplify regulatory T cell function [43, 44]. Thus, surgically injured tissues demonstrate proinflammatory responses, while leukocytes in blood stream become anti-inflammatory and hyporeactive [45]. Adaptation to surgical stress involves coordinating local inflammation with systemic anti-inflammation so as to allow the concentration of activated phagocytes and other effectors only at the injured local site [46]. The adequate presence of activate phagocytes and other effector cells at the injured site would be deemed critical. Thus, it is also important to understand the quantity of leukocytes available at the site.

The perioperative leukocyte distribution was studied in detail in adult patients (age 53 ± 15 years old, American Society of Anesthesiologists (ASA) Physical Score 1-3) undergoing general, urological, or orthopedic surgeries by Bartal et al. [47]. Neutrophil counts increased postoperatively, peaking at postoperative day 1 without any change in monocyte and B cell counts in this study. The numbers of various types of T cells were reduced with the lowest at 12 hours postoperatively. In another study of patients aged 18-90 years undergoing total hip replacement, neutrophil count was increased by 1.2-fold at 1 hour postoperatively [48]. At 24-hour after surgery, monocytes increased by 1.9-fold, different from the previous study. In contrast, CD4^+^ and CD8^+^ T cells were contracted to 0.77-fold and 0.71-fold, respectively, compatible with the previous study. Postoperative lymphopenia was also reported in the pediatric population, most profoundly at 12 hours after surgery, as in the case in adults [49, 50]. These distribution pattern of immune cell subsets in the perioperative period seems to be regulated at least in part by stress hormones [51]. Epinephrine and norepinephrine induce a redistribution of neutrophils, monocytes, and T cells from the marginated pool such as spleen, lungs, lymph nodes, and bone marrows into the bloodstream and temporarily increase blood leukocyte counts [52]. Then, cortisol induces the movement of monocytes and T cells out of the blood stream to the surgical site or back to their origins. In contrast, neutrophil count continues to increase as a result of emergency granulopoiesis. Among leukocytes, the characteristics of monocyte has been favorably studied, particularly on their human leukocyte antigen (HLA)-DR surface expression. HLA-DR is a component of major histocompatibility complex (MHC) class II and involved in antigen presentation to T cells. Anti-inflammatory cytokine IL-10 induces an accumulation of MHC class II complexes in intracellular vesicles and reduces HLA-DR surface expression [53]. Postoperative reduction of HLA-DR surface expression on monocytes has been reported in adult and pediatric patients [48, 54, 55].

SSI is established within the first few hours after tissues are contaminated by bacteria [56]. Thus, it is intuitive that intraoperative leukocyte dysfunction can pose a significant impact on the occurrence of SSIs. However, there is a paucity of clinical research examining the relationship between perioperative leukocyte function and the development of SSIs. As described above, patient-related factors such as older age, diabetes, smoking, and obesity are significantly associated with SSIs [16, 57–59]. Many components of WHO-recommended SSI perioperative bundles supported by strong evidence are to reduce microbial spillage into the otherwise sterile tissues, while perioperative oxygen is to optimize host immune function (Table 1). Once bacteria contamination occurs, the host responds and attacks the invading microbes in conjunction with the help of antimicrobials. Weak immune system will not work adequately for infection control. Older age, diabetes, smoking, and obesity—all are considered to be associated with impaired immune functions. For example, the dysfunction of phagocytes (defective chemotaxis, bacterial killing, superoxide production, phagocytosis, etc.) is well described in diabetes [60–63]. Good glucose level control is likely important, as demonstrated in the study that higher glucose level was associated with impaired phagocytic activity in diabetic patients and a better glucose control over 5 days enhanced phagocytic activity [64]. Maintaining and/or enhancing immune functions perioperatively would be thus a must. Diabetes, smoking, and obesity are something that we can intervene preoperatively by controlling blood glucose, cessation of smoking, and weight management. Here, we will review the selection of anesthetic drugs as a potential approach to optimize perioperative host immunity to reduce SSIs. A consideration of drug selection for anesthesia to optimize immune functions for SSIs is not a part of WHO recommendations. However, the importance of anesthetic drug selection has been intensively debated in the setting of cancer surgery [65–69]. In the following section, we review the update-to-date knowledge on the selection of anesthetic drugs from the standpoint of SSIs.

## 5. Anesthesia and SSIs

Anesthesia is largely categorized into general anesthesia and regional anesthesia. General anesthesia has been administered in a large number of surgical procedures. This is largely accomplished by intravenous anesthetics and/or volatile anesthetics. While propofol and etomidate, representative intravenous anesthetics, target *γ*-aminobutyric acid A (GABA_A_) receptor for their anesthetic effect [70–72], volatile anesthetics presumably target a number of receptors in the central nervous system (CNS) such as GABA_A_ receptor, N-methyl-D-aspartate (NMDA) receptor, and the two-pore domain potassium (K2P) channel [73]. Promiscuity of volatile anesthetics is well reflective in their effective concentrations; while propofol and etomidate work at the concentration of <10-50 *μ*M, volatile anesthetic effective concentrations are around 0.3-1 mM. Usually, multiple drugs are given intraoperatively for anesthesia care. Furthermore, patient population can be quite heterogeneous. Thus, determining whether or not a certain anesthetic drug affects immune cell functions would be practically a very difficult task. In addition, the majority of perioperative, immunological studies so far have been focusing on measuring the level of serum proinflammatory and anti-inflammatory cytokines [74], much less on characterizing leukocyte profiles. Neutrophils occupy 50-60% of the total circulating leukocytes in humans and are critical, first-defense phagocytes. Their number increases in the perioperative period, which makes them the major phagocytes. Thus, understanding neutrophil functions under different anesthetic drugs would be of particular interest. Because of issues of polypharmacy and patient heterogeneity in clinical setting as described above, the use of animal models as well as *in vitro*/*ex vivo* experiments should be used complimentarily to answer the question.

### 5.1. The Effect of Anesthetics on Neutrophil Functions and Infection In Vitro, In Vivo, and Ex Vivo

Commonly used volatile anesthetics are isoflurane, sevoflurane, and desflurane, all of which are derived from ether. The majority of studies have been done using either isoflurane or sevoflurane. Neutrophils have a number of functions as phagocytes including chemotaxis/recruitment and phagocytosis. Neutrophil recruitment to the site of infection is critical for microbial eradication. The study using the murine reverse Arthus reaction model, a well-known skin inflammation model, showed that 2% isoflurane exposure (both 2- and 4-hour exposure) reduced the number of recruited neutrophils by approximately 90% compared to no isoflurane exposure group, indicating that isoflurane had a significant impact on neutrophil mobility [75]. The effect of anesthetics on phagocytosis was evaluated *in vitro* using isoflurane, sevoflurane, and intravenous anesthetic propofol [76]. The study showed that both isoflurane and sevoflurane attenuated phagocytosis, but propofol did not. Phagocytosis was further tested *ex vivo* in pediatric patients who underwent cardiac catheterization either under volatile anesthetics or intravenous anesthetics by Koutsogiannaki et al. [77]. Volatile anesthetics attenuated neutrophil phagocytosis, but intravenous anesthetics did not. The role of anesthetics in bacterial loads was tested in the experimental polymicrobial sepsis model and the surgical wound infection model. For the former, mice underwent cecal ligation and puncture surgery under ketamine/xylazine anesthesia to induce polymicrobial abdominal sepsis, and a group of them further received 1% isoflurane either for 2 or 6 hours [78]. Neutrophils in mice that received 1% isoflurane for 2 or 6 hours demonstrated the attenuation of both the recruitment to the peritoneal cavity and the phagocytosis compared to mice without isoflurane exposure. However, bacterial loads were significantly increased only in mice exposed to isoflurane for 6 hours compared to mice without isoflurane exposure, which would be presumably due to the very short doubling time (20~ 30 min) of some of bacteria. Two-hour of suppression of neutrophil functions would not be long enough to significantly affect bacteria loads compared to 6-hour exposure. The result of bacterial loads was associated with the survival of mice; 6-hour isoflurane exposure worsened their survival significantly compared to mice without isoflurane exposure, while mice exposed for 2 hours did not show any difference in their survival. In the deep wound infection model, mice were subjected to the inoculation of MRSA strain USA300 in the deep thigh wound under ketamine/xylazine anesthesia, and some of them were further randomized into no-anesthesia, 2-hour,- or 6-hour anesthesia [77]. As anesthesia, isoflurane or propofol was given. While 2-hour isoflurane exposure did not affect bacterial loads, 6-hour isoflurane exposure significantly worsened bacterial loads, similar to the sepsis study. In contrast, both 2- and 6-hour propofol exposures did not affect bacterial loads. These results serve as a strong foundation to consider the possibility that the type of anesthetic drugs affects the occurrence of SSIs. Then, can we tell the underlying mechanism of volatile anesthetic-induced neutrophil functional impairment, if present? A number of direct anesthetic targets on immune cells have been reported. These targets include adhesion molecules integrins [79–84], TLRs [85–87], and intracellular molecule Rap1 [76]. Volatile anesthetic integrin targets include leukocyte-function associated antigen-1 (LFA-1) and macrophage-1 antigen (Mac-1), both of which are expressed on neutrophils. LFA-1 is critical for neutrophil recruitment and Mac-1 is critical for microbial phagocytosis [88, 89]. TLR2 and TLR4 are critical for gram-positive and gram-negative bacterial components. Rap1 functions for phagocytosis. Thus, inhibition of their functions by volatile anesthetics in concert is considered to contribute to the impairment of neutrophil functions.

### 5.2. Clinical Studies regarding Anesthetic Drugs and Surgical Site Infections

While a large number of studies have examined the relationship between anesthetic drugs and cancer resection surgery [90–92], the topic on the relationship between anesthetic drugs and surgical site infections has been less investigated. A study by Chang et al. used the Longitudinal Health Insurance Database of Taiwan to identify 3,081 patients (mean age 62.6 years old) who underwent total hip or knee replacement from 2002 to 2006 and compared the incidence of SSIs between patients who received general anesthesia and ones who received either epidural or spinal anesthesia [93]. They found that patients who underwent general anesthesia had SSI incidence of 2.8%, while patients who received epidural or spinal anesthesia had SSI incidence of 1.2%. After adjusting for patient's age, sex, comorbidities, surgery type, surgeon's age, and hospital teaching status, the odds of SSIs for patients under general anesthesia were 2.21 times higher. Plausible mechanisms were not described, however. Over the years, other studies also have examined a variety of outcomes for total hip or knee replacement under different anesthetic regimens. Johnson et al. performed the meta-analysis of 29 studies involving 10,488 patients and showed that there was no difference in SSIs between general anesthesia and spinal/epidural anesthesia [94]. The incidence of SSIs in total knee or hip replacement is not high, around 2% [95]. The studies above did not describe the operative time, but other studies indicated <2 hours [96, 97].

Certainly, surgical procedures associated with much higher incidences of SSIs would be important procedures to evaluate the role of anesthetic drugs in SSIs. Surgical wound for total knee or hip replacement is usually considered clean wound. Clean-contaminated wound such as wounds in gastrointestinal surgeries would certainly fit for this purpose. Koo et al. performed retrospective study of 1,934 patients (mean age around 60 years old) who underwent colorectal surgery under volatile anesthetics or propofol-based intravenous anesthesia from 2011 to 2013 [98]. They found that patients receiving volatile anesthetics (sevoflurane or desflurane) had 2.5% of SSIs, while patients in propofol arm had 0.5% of SSIs. After 1 : 1 propensity score matching, volatile anesthetics arm and propofol arm had 2.6% and 0.5% of SSIs, respectively. In this cohort, the ratio of open procedures to laparoscopic procedures was roughly 1 : 1. The average anesthetic time was around 3.5 hours. Shimizu et al. retrospectively examined 265 patients (average 68 years old) who underwent open gastrointestinal surgeries under sevoflurane anesthesia or propofol anesthesia from 2007 to 2008 [99]. Sevoflurane and propofol arms did not show any difference in SSIs (10.5% vs. 11.2%, respectively). However, after 1 : 1 propensity score matching (each arm 84 patients), sevoflurane arm had 7.1% of SSIs, while propofol arm had 16.7%. The low number of patients assigned to each arm following matching might have significantly affected the result in this study. We also examined the risk factors of SSIs in 621 pediatric patients (average age of 8.8 years) undergoing gastrointestinal surgery from 2017 to 2019 [100]. These surgeries were performed under our institutional SSI bundle, and SSIs were prospectively examined. The majority of patients' wound class was classified as clean-contaminated. SSIs were seen in 6.2% of cases. All the patients underwent general anesthesia with volatile anesthetics (the majority received sevoflurane). Although the duration of surgery did not differ between patients with and without SSIs, total sevoflurane dose was higher in patients with SSIs. After dichotomizing the patients by median total sevoflurane dose, patients were 1 : 1 propensity matched including patient demographics, American Society of Anesthesiologist (ASA) Physical score, wound classes, preoperative prep, prophylactic antibiotics, and surgeons. 9.8% of higher sevoflurane dose group had SSIs, while 3.9% of lower sevoflurane dose group had SSIs. 70% had open procedures. Two groups' anesthetic duration was not different (median ~ 300 minutes). Yamamoto et al. recently reported the study of SSIs of 326 patients (median age 70 years old) who underwent pancreaticoduodenectomy (median duration of surgery 10.9 hours) under general anesthesia between 2009 and 2018 [101]. 18.4% of patients developed SSIs. The multivariable analysis showed that the use of desflurane was associated with a significantly lower risk of SSI than sevoflurane (odds ratio 0.503), though authors did not perform propensity-matching. So far, the limited study has been done on the effect of desflurane on immune targets, and it would be extremely important to delineate desflurane's property in the future. The animal studies demonstrated that the duration of anesthesia exposure played a role in the degree of infection. Although all the studies did not assess perioperative immune functions, the duration of surgery/anesthesia exposure time was in line with the animal data assessing infection and anesthetic duration as described above [77, 78].

We also examined the risk factors of SSIs in pediatric patients who underwent cerebral spinal fluid conversion procedures for hydrocephalus (Shibamura-Fujiogi et al., under review). The majority of wounds were classified as clean, and SSIs were seen in 3.6%. The median duration of anesthesia was 170 minutes. We did not find any difference in SSIs based on the dose of volatile anesthetics, in line with the animal data that the short exposure of volatile anesthetic did not change bacterial loads. We also examined SSIs in spine surgery, where the majority of wounds were considered clean. The incidence of SSIs was 1.4%. The majority of the SSIs were from the deep wounds, where a large number of bacteria were slow growing species (Serratia, Proteus, Bacteroides, Enterobacter, Morganella, etc.). Spine fusion was primarily anesthetized by intravenous anesthetics.

## 6. Future Direction/Practical Suggestion

In the future, it will be important to continue to examine the role of anesthetic drug selection in high-risk SSI surgery such as gastrointestinal surgery. Duration of anesthesia may be one of the important factors. In addition, it will be important to determine if only a certain types of procedures are relevant from SSI standpoint. Continuous investigation will provide us an important information about the role of anesthetics in SSIs.

## Figures and Tables

**Table 1:** Strong recommendations by WHO.

Preoperative
(i) Decolonization with mupirocin ointment with or without chlorhexidine gluconate body wash in nasal carriers of Staphylococcus aureus undergoing cardiothoracic and orthopedic surgery.
(ii) Mechanical bowel preparation without the use of oral antibiotics
(iii) Hair removal
(iv) Prophylactic antibiotic should be administered within 120 minutes before incision, while considering the half-life of the antibiotic.
(v) Surgical hand preparation should be performed either by scrubbing within a suitable antimicrobial soap and water or using a suitable alcohol-based hand rub before donning sterile gloves.
(vi) Alcohol-based antiseptic solutions based on chlorohexidine (CHG) for surgical site skin preparation should be used in patients undergoing surgical procedures.
Intraoperative/postoperative
(i) Perioperative oxygenation
(ii) Surgical antibiotic prophylaxis administration should not be prolonged after completion of the operation.

## Data Availability

All the data are available in the manuscript.
